# Salivary gland ultrasound abnormalities in primary Sjögren’s syndrome: consensual US-SG core items definition and reliability

**DOI:** 10.1136/rmdopen-2016-000364

**Published:** 2017-06-09

**Authors:** Sandrine Jousse-Joulin, Emmanuel Nowak, Divi Cornec, Jackie Brown, Andrew Carr, Marina Carotti, Benjamin Fisher, Joel Fradin, Alojzija Hocevar, Malin V Jonsson, Nicoletta Luciano, Vera Milic, John Rout, Elke Theander, Aaltje Stel, Hendrika Bootsma, Arjan Vissink, Chiara Baldini, Alan Baer, Wan Fai Ng, Simon Bowman, Zarrin Alavi, Alain Saraux, Valérie Devauchelle-Pensec

**Affiliations:** 1 Department of Rheumatology, Cavale Blanche Hospital and Brest Occidentale University, Brest, France; 2 INSERM CIC 1412, Brest Medical University Hospital, Brest, France; 3 Department of Dental and Maxillofacial Radiology, KCL Dental Institute, Guy’s Hospital, London, UK; 4 Dental Radiology, Dental Hospital, Newcastle upon Tyne Hospitals NHS Foundation Trust, London, UK; 5 Department of Radiology, Polytechnic University of the Marche, Ancona, Italy; 6 Rheumatology Research Group, Institute of Inflammation and Ageing, University of Birmingham, Birmingham, UK; 7 Department of Rheumatology, University Hospitals, Birmingham NHS Trust, Birmingham, UK; 8 Department of Imaging, Johns Hopkins Bayview Medical Center, Baltimore, USA; 9 Department of Rheumatology, University Medical Centre, Ljubljana, Slovenia; 10 Department of Clinical Dentistry, Section for Oral and Maxillofacial Radiology, University of Bergen, Bergen, Norway; 11 Rheumatology Unit, University of Pisa, Pisa, Italy; 12 Institute of Rheumatology, School of Medicine, University of Belgrade, Belgrade, Serbia; 13 Department of Radiology, Birmingham Dental Hospital, St Chad’s Queensway, Birmingham, UK; 14 Department of Rheumatology, Skane University Hospital, Malmö, Sweden; 15 Department of Rheumatology and Clinical Immunology, University of Groningen, University Medical Centre Groningen, Groningen, The Netherlands; 16 Department of Oral and Maxillofacial Surgery, University of Groningen, University Medical Centre Groningen, Groningen, The Netherlands; 17 Department of Medicine (Rheumatology), Johns Hopkins University School of Medicine, Baltimore, USA; 18 Institute of Cellular Medicine, Newcastle University & NIHR Newcastle Biomedical Research Centre, Tyne, UK

**Keywords:** Ultrasound, Salivary glands, Primary Sjögren’s syndrome, Atlas, Classification criteria

## Abstract

**Objectives:**

Ultrasonography (US) is sensitive for detecting echostructural abnormalities of the major salivary glands (SGs) in primary Sjögren’s syndrome (pSS). Our objectives were to define selected US-SG echostructural abnormalities in pSS, set up a preliminary atlas of these definitions and evaluate the consensual definitions reliability in both static and acquisition US-SG images.

**Methods:**

International experts in SG US in pSS participated in consensus meetings to select and define echostructural abnormalities in pSS. The US reliability of detecting these abnormalities was assessed using a two-step method. First 12 experts used a web-based standardised form to evaluate 60 static US-SG images. Intra observer and interobserver reliabilities were expressed in κ values. Second, five experts, who participated all throughout the study, evaluated US-SG acquisition interobserver reliability in pSS patients.

**Results:**

Parotid glands (PGs) and submandibular glands (SMGs) intra observer US reliability on static images was substantial (κ > 0.60) for the two main reliable items (echogenicity and homogeneity) and for the advised pSS diagnosis. PG inter observer reliability was substantial for homogeneity. SMGs interobserver reliability was moderate for homogeneity (κ = 0.46) and fair for echogenicity (κ = 0.38). On acquisition images, PGs interobserver reliability was substantial (κ = 0.62) for echogenicity and moderate (κ = 0.52) for homogeneity. The advised pSS diagnosis reliability was substantial (κ = 0.66). SMGs interobserver reliability was fair (0.20< κ ≤ 0.40) for echogenicity and homogeneity and either slight or poor for all other US core items.

**Conclusion:**

This work identified two most reliable US-SG items (echogenicity and homogeneity) to be used by US-SG trained experts. US-PG interobserver reliability result for echogenicity is in line with diagnosis of pSS.

**Key messages**

**What is already known about this subject?**

- Diagnosis of primary Sjögren’s syndrome (pSS) is a challenge.
- Ultrasound of salivary glands (US-SG) is a valuable diagnostic tool.
- Yet there is no gold standard of US diagnosis for echostructural abnormalities in pSS.

**What does this study add?**

- This work put forward a preliminary atlas of echostructural abnormalities in pSS.
- This work identified two most reliable US-SG items: echogenicity and homogeneity.

**How might this impact on clinical practice?**

- Trained US-SG experts can use our preliminary atlas of US-SG abnormalities.
- This can be done concomitantly with other classification criteria in diagnosis of pSS.

## Introduction

Lymphocytic infiltration of the salivary glands (SGs) is a key pathological feature of primary Sjögren’s syndrome (pSS).^1 2^ Currently available tools for assessing the SGs include salivary flow measurement, minor SG biopsy, sialography, scintigraphy, CT and MRI. Ultrasonography (US) was introduced more recently.^3–7^ US holds considerable appeal, as it is non-invasive, does not involve ionising radiation, can be repeated many times and is available as an outpatient investigation. Both researchers and clinicians have identified US as a valuable tool for diagnosing pSS^8–15^ and as a potential source of classification criteria for this disease. Moreover, the advent of new treatments for pSS^16^ has created a need for valid and easy-to-use imaging tools capable of detecting changes in disease activity over time.^17^ Using the Outcome Measures in Rheumatology (OMERACT) filter,^18^ we recently reported the need for a consensual scoring system, SG US expert training as well as evaluation of US criterion validity, that is, comparing minor SG biopsy to minor SG US.^19^ The review results were consistent with those of the literature^20–25^ and our review is the first step in setting up an OMERACT standard that will open further avenues for the use of such promising diagnostic tool.^26^ Today, US cannot yet be used as the sole diagnostic bedside tool in pSS but as an early diagnostic tool when used carefully by experienced US experts.

Here, our objectives were to define selected US-SG echostructural abnormalities in pSS, set up a preliminary atlas of these definitions and evaluate the consensual definitions reliability in both static and acquisition US-SG images.

## Method

### Definition of the core items of ultrasound SGs

During the 2012 American Congress of Rheumatology meeting, international pSS experts (from France, Norway, Italy, England, Serbia, Slovenia, Sweden, The Netherlands and USA) who had at least 5 years of experience with US in pSS were invited to work on the study. Among 10 experts, only 6 contributed to the first meeting towards achieving a consensus definition of US-SG abnormalities in pSS. They selected a preliminary core set of US-SG items worthy of routine evaluation. These experts then completed an email questionnaire, indicating whether they agreed with each definition (yes/no answers). The same six experts followed up to the 2013 and 2014 European League of Rheumatism (EULAR) meetings where preliminary 2012 meeting results were presented.

### Set-up of a SG ultrasound atlas

Finally, in 2014 consensus was reached in regard with the US-SG core items and a preliminary atlas was set forth.

This initial atlas included only consensual B-mode images in pSS and will be used by the experts.

### Reliability of US-SG core items on static US images

Twelve experts performed a web-based standardised form (table 1) to evaluate 60 static images (30 parotid glands (PGs) and 30 submandibular glands (SMGs)) (Philips iU22, 12.5 MHz linear array transducer; Philips Healthcare, Amsterdam, The Netherlands). The set of B-mode images (with the same setting) of the major SGs, from Brest centre data bank, of patients with pSS and normal individuals was chosen in order to have both images of normal (healthy individuals) and abnormal SG parenchymal echostructure (pSS patients) and were sent to other centres. Each expert evaluated the items selected by consensus (ie, the preliminary atlas) in two rounds, at an interval of 3 months. Then an advice for pSS diagnosis (rule out, rule in, indeterminate) was given by the experts. The results were used to assess interobserver and intraobserver reliabilities. All images were read anonymously and in random order.

**Table 1:** Standardised form used to assess the reliability of ultrasound of salivary gland core items of the images

	Parotid glands		Submandibular glands	
	Right	Left	Right	Left
Echogenicity				
Normal, (0); Abnormal (fibrosis), 1				
Homogeneity				
Normal, 0; Abnormal, 1				
Hyperechoic bands				
None (0), <50% of the parenchyma (1), ≥50% (2)				
Number of hypoechoic/anechoic areas*				
Size of the largest hypoechoic/anechoic area (mm)*				
Location of the hypoechoic/anechoic areas in the gland				
None (0), isolated (<25% of the surface area) (1), localised (25%–50%) (2), scattered (>50%) (3), diffused (4)				
Number of abnormal lymph nodes in the glands*				
Presence of normal lymph nodes at the upper and/or lower poles of the parotid glands: no (0), yes (1)				
Calcifications				
No (0), yes (1)				
Posterior border visible				
No (0), yes (1)				
Diagnosis advice of pSS based on the seven items				
ruled out (0), indeterminate (1), ruled in (2)				

*Quantitative variables were then categorised as follows: number of hypoechoic/anechoic areas: None (0), 1–4 (1), ≥5 (2); size of the largest area: None (0), ≤2 (1), >2 (2); abnormal lymph nodes: No (0), Yes (1).

pSS, primary Sjögren’s syndrome.

### Reliability of US-SG core items on acquisition imaging in patients with pSS

Five experts, of the initial six who participated in development of the definitions and the atlas, participated in assessment of the reliability of the consensual items in acquisition imaging. Over a 2-day period, each expert performed US of both PGs and both SMGs of 19 patients with pSS (with or without known SG abnormalities). Various US machines were used (Mylab ALPHA, Mylab60 and Mylab 6; all from Esaote, Genoa, Italy). Given the difference in each patient’s SG echogenicity US B-mode settings were adjusted according to each patient. The time needed for each expert to examine the 19 patients was recorded. Approval was obtained from Brest ethics committee and the study was referred in clinical trial (NCT 02358213).

### Statistical analysis

Cohen’s κ was used to measure interobserver agreement for binary items. Weighted κ (Fleiss-Cohen weights) was used for items with more than two ordinal categories.^27^ κ coefficients were calculated for each pair of observers, leading to mean value, minimum and maximum for each item. The same coefficients were calculated for each observer to measure intraobserver agreement between the first and the second interpretation, leading to mean value, minimum and maximum for each US-SG core item.^28^

Number of hypoechoic or anechoic areas was recorded as: none, 1–4 or ≥5 and location was reported as follows: none, isolated, localised, scattered or diffuse. Number of abnormal lymph nodes was recorded as present or absent. Diagnosis advice was reported as follows: ruled out, indeterminate or ruled-in.

According to Landis and Koch,^29^​  κ values were interpreted as follows: <0.00 poor, 0.00–0.20 slight, 0.21–0.40 fair, 0.41–0.60 moderate, 0.61–0.80 substantial and 0.81–1.00 almost perfect.

## Results

### Definition of US-SG echostructural abnormalities (US-SG core items)

**Figure 1:**
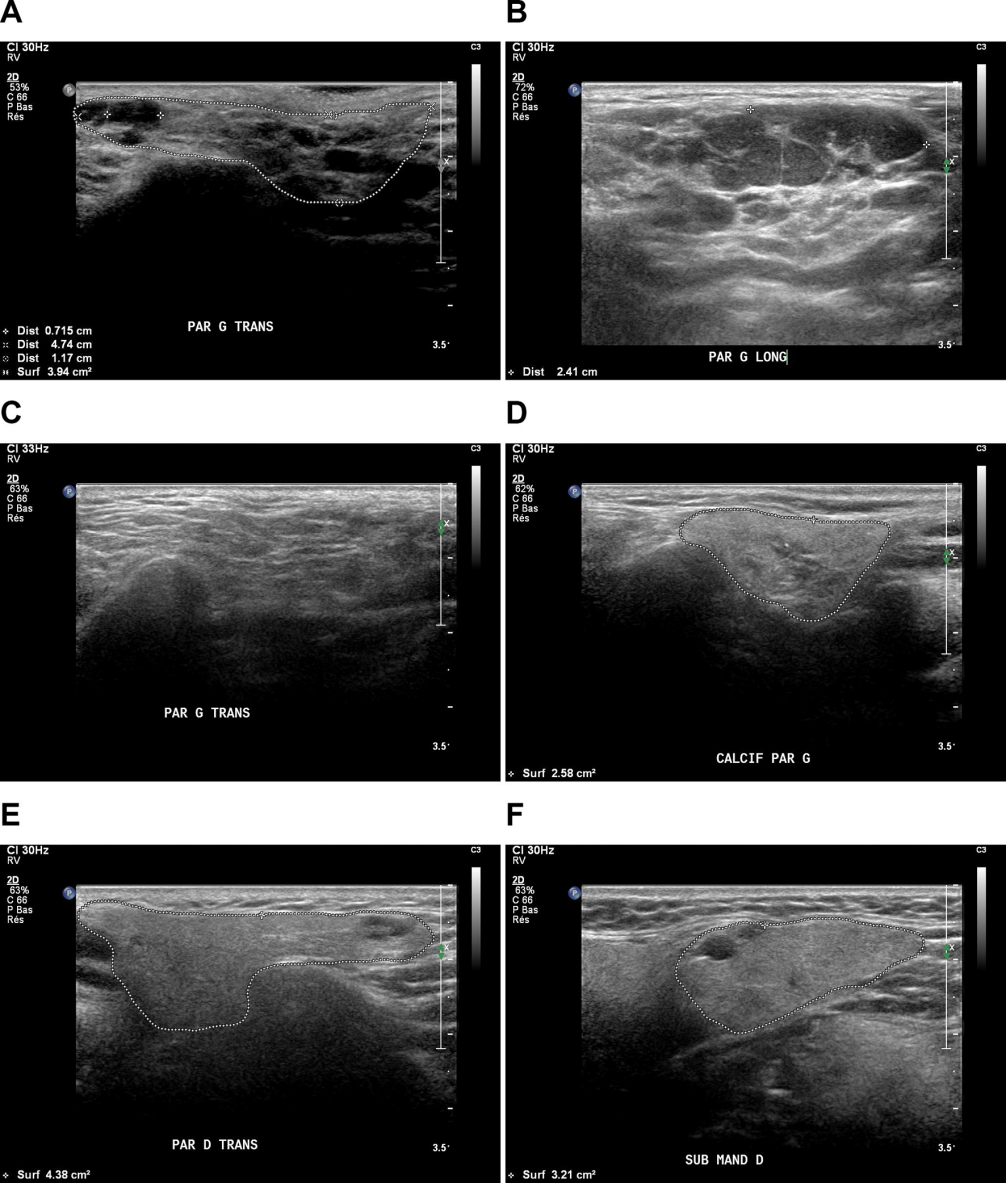
The  core items were (A) hypoechogenicity, (B) heterogeneity (numerous anechoic areas), (C) hyperechoic bands, (D) calcifications (star), (E) lymph nodes (white arrow), (F) anechoic area, (A, D,E,F) posterior border (dotted line).

The experts selected the US-SG echostructural abnormalities related to: echogenicity, homogeneity, lymph nodes, posterior border, in B-mode (see online supplementary table 1). The definitions developed during the first meeting were modified during the second meeting (see online supplementary table 1). Complete agreement was reached about the following items definitions: echogenicity, homogeneity, lymph nodes, posterior border, calcification, hyperechoic bands, hypoechoic/anechoic areas, location of hypoechoic/anechoic areas and abnormal lymph nodes. We called these items US-SG core items. An initial consensual reference atlas (33 consensual images) was developed based on these definitions (figure 1 and see online supplementary atlas).

10.1136/rmdopen-2016-000364.supp1 Supplementary data

10.1136/rmdopen-2016-000364.supp2 Supplementary data

### Reliability of SG ultrasound core items using static images

#### Intraobserver reliability

PG intraobserver US reliability was substantial for: echogenicity, homogeneity, number and location of hypoechoic/anechoic areas, and normal lymph nodes. The advised pSS diagnosis reliability was substantial (κ=0.86) (table 2). In contrast, intraobserver reliability was moderate for hyperechoic bands, number of abnormal lymph nodes, and fair for calcifications, posterior border visibility.

**Table 2:** Intraobserver and interobserver reliability of static ultrasound core items of PG and SMG: κ values

	Echogenicity	Homogeneity	Hyperechoic bands	Number of hypoechoic/anechoic areas*	Location of hypoechoic/anechoic areas	Number of abnormal nodes*	Normal nodes	Calcifications	Posterior border visibility	Diagnosis advice†
PG intraobserver										
Mean	0.75	0.75	0.58	0.80	0.78	0.47	0.66	0.38	0.37	0.86
Min	0.43	0.29	0.00	0.57	0.59	0.00	0.44	−0.03	−0.11	0.52
Max	0.93	1	1	0.92	0.94	1	0.90	1	0.80	1
SMG intraobserver										
Mean	0.61	0.63	0.61	0.76	0.69	0.17	0.45	0.42	0.41	0.70
Min	0.23	0.25	0.36	0.54	0.45	0.00	0.00	−0.05	0.00	0.33
Max	1	0.93	1	1	0.92	0.84	1	0.89	0.91	0.92
PG interobserver										
Mean	0.60	0.66	038	0.74	0.77	0.19	0.54	0.10	0.15	0.78
Min	0.16	0.12	0.00	0.39	0.44	−0.06	0.25	−0.05	−0.38	0.31
Max	0.93	1	0.88	0.96	0.94	1	0.90	0.65	0.59	0.98
SMG interobserver										
Mean	0.38	0.46	0.32	0.53	0.52	0.01	0.09	0.26	0.15	0.54
Min	0.01	0.01	0.00	0.22	0.07	−0.06	−0.11	0.00	−0.09	0.16
Max	0.92	0.93	0.79	0.90	0.92	0.27	0.67	1	0.65	0.91

*Quantitative variables were then categorised as follows: number of hypoechoic/anechoic areas: None (0), 1–4 (1), ≥5 (2); abnormal lymph nodes: No (0), Yes (1).

†Diagnosis advice was given in regard with the typical pattern of pSS on images and not in patients with pSS.

PG, parotid gland; pSS, primary Sjögren’s syndrome; SMG, submandibular gland.

SMG intraobserver US reliability was substantial for echogenicity, homogeneity, hyperechoic band, hypoechoic/anechoic areas and location. The results were moderate for normal lymph nodes, calcification and posterior border. The advised diagnosis reliability was substantial (κ=0.70).

#### Interobserver reliability

PG interobserver reliability was substantial for homogeneity, number and location of hypoechoic/anechoic areas and moderate for echogenicity and normal lymph nodes. The results were fair for hyperechoic bands and slight for calcification and posterior border. The advised diagnosis reliability was substantial (κ=0.78). SMG interobserver reliability was fair for echogenicity and calcification. The results were: moderate for homogeneity, hypoechoic/anechoic areas and location; slight for posterior border and normal lymph nodes. Advised diagnosis reliability was moderate (κ=0.54).

### Distribution of ultrasound core items for static images

Item distributions were similar between the two readings. Echogenicity was regarded as normal in >50% of both PGs and SMGs. Homogeneity was regarded as abnormal in >50% of the SMGs and 48.6%–52.1% of the PGs. Hyperechoic bands occupying <50% of the gland surface area of both PGs and SMGs were seen in >50% of the cases (table 3). In all four SGs, hypoechoic/anechoic areas were not found in 44.9%–53.1% of cases, and ≥5 hypoechoic areas were found in 23.9%–33.0% of the cases. Normal-appearing lymph nodes were seen in >20% of PGs and <10% of SMGs. Abnormal lymph nodes and calcifications were rare in both PGs and SMGs. The posterior border was usually not visible, particularly for the SMGs.

**Table 3:** Distribution of ultrasound items for static images

	Parotid glands		Submandibular glands	
	First reading	Second reading	First reading	Second reading
Echogenicity				
Normal	58.9%	57.4%	59.6%	58.1%
Abnormal	41.1%	42.6%	40.4%	41.9%
Homogeneity				
Normal	51.4%	47.9%	47.5%	45.1%
Abnormal	48.6%	52.1%	52.5%	54.9%
Hyperechoic bands				
None	36.1%	36.5%	42.8%	41.5%
<50%	51.9%	52.1%	54.2%	52.7%
≥50%	11.9%	11.4%	3.1%	5.9%
Number of hypoechoic/anechoic areas				
None	53.1%	48.2%	49.7%	44.9%
1–4	14.2%	18.8%	26.4%	28.5%
≥5	32.7%	33%	23.9%	26.6%
Location of hypoechoic/anechoic areas				
None	51.4%	47.3%	49.0%	44.7%
Isolated	11.9%	14.8%	21.2%	20.8%
Localised	10.0%	16.4%	12.5%	14.9%
Scattered	6.4%	3.9%	5.0%	4.8%
Diffuse	20.3%	17.5%	12.3%	14.9%
Normal nodes				
No	73.6%	73.3%	92.5%	93.6%
Yes	26.4%	26.7%	7.5%	6.4%
Abnormal nodes				
No	93.3%	94.7%	98.0%	98.6%
Yes	6.7%	5.3%	2.0%	1.4%
Calcifications				
No	86.1%	86.6%	83.8%	86.0%
Yes	13.9%	13.4%	16.2%	14.0%
Posterior border visible				
No	41.9%	45.0%	17.0%	22.0%
Yes	58.1%	55.0%	83.0%	78.0%
pSS diagnosis advice*				
Ruled out	55.8%	51.4%	55.8%	50.4%
Indeterminate	9.7%	12.8%	11.9%	16.8%
Ruled in	34.4%	35.7%	32.2%	32.8%

*Diagnosis advice was given in regard with the typical pattern of pSS on images and not in patients with pSS.

pSS, primary Sjögren’s syndrome.

### Reliability of SG ultrasound core items on acquisition imaging

PGs interobserver reliability was substantial for echogenicity and moderate for homogeneity, number and location and the size of the largest hypoechoic/anechoic area but fair for hyperechoic bands and slight for normal lymph nodes, number of abnormal lymph nodes, calcification and posterior border visibility (table 4). The advised diagnosis reliability of pSS was substantial (κ=0.66). SMGs interobserver reliability was fair for echogenicity, homogeneity and number and location of hypoechoic/anechoic areas. The results were slight for normal lymph nodes, calcifications and posterior border visibility. Advised diagnosis reliability was fair (κ=0.38).

As shown in table 5, mean time duration of US ranged across observers from 11 to 27 min.

**Table 4:** Interobserver reliability of image acquisition: κ values for ultrasound items in the parotid and submandibular glands

Item	PG	SMG
Echogenicity		
Mean	0.62	0.28
Min–Max	0.42–1.00	0.00–0.58
Homogeneity		
Mean	0.52	0.26
Min–Max	0.16–0.87	−0.05 to 0.87
Hyperechoic bands		
Mean	0.38	0.19
Min–Max	0.11–0.64	−0.09 to 0.39
Number of hypoechoic/anechoic areas*		
Mean	0.54	0.27
Min–Max	0.34–0.88	0.09–0.51
Size of the largest hypoechoic/anechoic area*		
Mean	0.41	0.17
Min–Max	0.29–0.79	0.04–0.41
Location of hypoechoic/anechoic areas		
Mean	0.58	0.30
Min–Max	0.26–0.85	0.08–0.65
Number of abnormal lymph nodes *		
Mean	–	–
Min–Max	–	–
Normal lymph nodes		
Mean	0.13	0.01
Min–Max	−0.07 to 0.28	−0.09 to 0.26
Calcifications		
Mean	0.03	0.03
Min–Max	−0.05 to 0.37	−0.10 to 0.19
Posterior border visibility		
Mean	0.02	0.15
Min–Max	−0.16 to 0.18	0.00–0.48
Diagnosis advice*		
Mean	0.66	0.38
Min–Max	0.30–0.97	0.12–0.65

Quantitative variables were then categorised as follows: number of hypoechoic/anechoic areas: None (0), 1–4 (1),≥5 (2); size of the largest area: None (0), ≤2 (1), >2 (2); abnormal lymph nodes: No (0), Yes (1).

*Diagnosis advice was given in regard with the typical pattern of pSS on images and not in patients with pSS.

PG, parotid gland; pSS, primary Sjögren’s syndrome; SMG, submandibular gland.

**Table 5:** Time in minutes needed by each observer to perform salivary gland ultrasonography

Observer	Min	Mean	Median	Max
A	8	11.4	11	16
B	16	27	26	36
C	15	23	23	40
D	7	12	12	15
E	13	18.8	18	27

## Discussion

Given the multitude of US-SG abnormalities in pSS, it has become a challenge to reach consensus on the definition and scoring of the most reliable US-SG abnormalities.^19^ We conducted this study to develop an international consensus about the definitions of echostructural abnormalities of SG in pSS and to evaluate the reliability of US in detecting them.

Homogeneity and echogenicity items showed substantial intraobserver reliability for both PGs and SMGs on static images. Whereas interobserver reliability of homogeneity was only substantial in PGs and moderate in SMGs and that of echogenicity was moderate in PGs and fair again in SMGs. Heterogeneity was defined as the presence of hypoechoic/anechoic areas with or without hyperechoic bands. For both glands, there is an abundance of hypoechoic/anechoic areas in pSS and their absence in normal individuals. However, the presence of highly vascularised fatty infiltration (ie, SMG echotexture vs PG echotexture) in SMGs may contribute to the difference observed in interobserver reliabilities of homogeneity item. These findings are consistent with those reported by Yoshiura *et al*.^7^

In line with previous studies,^7 30^ several core items showed low reliability in our study, namely, hyperechoic bands, calcifications and posterior border visibility for PGs and SMGs. Even though hyperechoic bands were defined by consensus, their reliable assessment in pSS remains challenging. Hyperechoic bands may develop in normal individuals due to advanced age or fibrosis of the SGs.

The static image inter-reliability of lymph nodes was moderate for the PGs but only slight for the SMGs, a finding that may reflect the usual absence of visible lymph nodes in the SMGs^7 31^ and their usual presence at specific locations in the PGs. Despite the definition established by consensus, calcifications were also difficult to assess. This finding may be ascribable to technical factors: when using static images, image quality cannot be optimised (for instance by changing the scanned area) and calcifications may be mistaken for hyperechoic bands. None of the nine published studies on the interobserver reliability of SG US in pSS^7 10 15 20 25 30 32–35^ evaluated calcifications. The posterior border was also difficult to assess, particularly for PGs, due to their anatomy and location in the retromandibular fossa.^32^ In our study, both PGs and SMGs inter-reliability and intrareliability were moderate to substantial for echogenicity and homogeneity (echostructure) due to the presence and distribution of hypoechoic/anechoic areas. 

In the acquisition imaging study in 19 patients with pSS, homogeneity item showed moderate interobserver reliability for PGs and fair for SMGs. Whereas the results for echogenicity item of PG and SMG were substantial and fair, respectively. Our results contradict a single-centre study by Carotti *et al*,^10^ who reported better reliability for SMGs than for PGs. Nevertheless, moderate interobserver reliability for PG homogeneity and fair one for SMG were consistent with those of the literature.^36–38^

In our study, the experts’ advised diagnosis of pSS on static US images showed substantial interobserver and intraobserver reliability for PGs and intraobserver reliability for SMGs. Experts’ advised diagnosis of pSS on acquisition US showed substantial interobserver reliability for PGs and fair for SMGs. These results may be explained by the better echogenicity of PGs compared with SMGs. These findings can be ascribed to the development of a novel US atlas as a prerequisite to performing careful ultrasound evaluations by trained US experts of the SGs in patients with pSS.^39^

Our study had several limitations. First limitation was the small number of US experts and US acquisitions performed, which precluded a large-scale Delphi.^36 40^

However, pSS is not a so common disease^41 42^ and, consequently, few experts were trained in SG US at the time of the study. Second, different US machines were used for acquisition in pSS patients, that is, not all experts had prior training with the proposed machines. In addition, intraobserver reliability of US-SG core items was not evaluated due to the lack of time during our study. Another limitation was that the interobserver reliability of abnormal lymph nodes could not be evaluated yet can be explained by the rare nature of this US-SG item in pSS. Our study drawback is the fact that the images (both in static and in acquisition mode) were not characteristic of patients seen in consultation for a suspicion of pSS. The assessment of the abnormal echostructure (ie, typical pattern of an inhomogeneous gland with hypoechoic/anechoic areas in its parenchyma) of the images led to the ‘advised diagnosis’ by experts. We did not perform a diagnosis of pSS patients. The typical pattern is a conclusive characteristic of pSS but not present in all pSS patients.

In conclusion, this study is the first attempt to set forth a preliminary atlas of consensual US images and US-SG core items definitions. We assessed the reliability of US-SG core items of the images in regard with the typical pattern of an inhomogeneous gland with hypoechoic/anechoic areas in its parenchyma, (ie, not present in all pSS patients) to identify those most reliable. The reliability results of the pSS typical pattern on these images can be used carefully by US-SG trained experts and concomitantly with other classification criteria in diagnosis of pSS. US-PG interobserver reliability result (substantial) for echogenicity seems to be in line with that of advised diagnosis of pSS.

Larger sample US-SG acquisition studies using the same US machine by US-SG trained experts are warranted to validate our results and to further evaluate intraobserver reliability of US-SG items. Our US-SMG reliability results open the avenue to further search for other reliable and relevant abnormality definitions and scorings of SMG echotexture—to better distinguish them from neighbouring tissues.
