# Supplementary material for: Salivary gland ultrasound abnormalities in primary Sjögren’s syndrome: consensual US-SG core items definition and reliability
Source: RMD Open. 2017 Jun 9;3(1):e000364. doi: 10.1136/rmdopen-2016-000364 (PMC5575597; doi:10.1136/rmdopen-2016-000364)
Supplement: Supplementary data [file rmdopen-2016-000364supp001.docx]

**Supplementary file: Table S1.** Development of a consensus on definitions of ultrasound salivary gland normal and abnormal echostructures (US-SG core items**)**

| **Definitions of US-SG normal and abnormal echostructures** | Round 1 | Suggestions that were added and accepted | Round 2 |
| --- | --- | --- | --- |
| **Echogenicity**  *Normal echogenicity* of the SGs defined as identical to that of the normal thyroid gland. In older patients, involution and fibrosis of the SGs may result in atrophy and hyperechogenicity.  *Abnormal echogenicity:* Echogenicity of the SG parenchyma is decreased by the presence of hypo/anechoic areas and increased by fibrosis or fatty infiltration. Consequently, SGs containing high-density fibrous tissue should be described as hyperechoic. | 50% | Echogenicity varies along a continuum from hypoechogenicity (numerous hypo/anechoic areas) to hyperechogenicity (fatty infiltration or fibrosis with hyperechoic bands). Echogenicity could be also compared to the adjacent muscles and should be more echoic than muscles.  SG echogenicity depends on the amount of intraglandular fatty tissue. | 100% |
| **Homogeneity**  *Normal homogeneity* is similar to that of the normal thyroid gland.  *Abnormal homogeneity* or abnormal SG echostructure: patients with pSS typically have a heterogeneous echostructure of all major SGs. However, heterogeneity may not be obvious in patients with intraglandular cyst-like areas or hypo/anechoic areas that are few in number or irregularly distributed in the parenchyma. | 50% | Abnormal homogeneity is defined as:   - numerous hypo/anechoic areas - numerous hyperechoic bands - hypo/anechoic areas | 100% |
| **Hypo/anechoic area #**  Hypo/anechoic areas are defined as small areas located anywhere within the SG, not compressible by the probe, and generating few or no echoes. These areas have no blood flow detectable by colour Doppler. Their centre is not hyperechoic. | 66% | Whenever possible, measure the largest dimension of hypo/anechoic area | 100% |
| **Normal Lymph nodes**  A lymph node is defined as an anechoic, round or oval area, with or without an echogenic hilus and with or without blood flow by colour Doppler. Lymph nodes are usually avascular and less than 1 cm in diameter. | 83% | In PGs: lymph nodes are located in the upper and lower poles of the gland or, occasionally, in the middle of the superficial portion of the gland. Normal SMGs do not contain any lymph nodes visible by ultrasonography. | 100% |
| **Calcifications***  Calcifications are defined as hyperechoic structures with or without acoustic shadowing, located within the parenchyma or ducts. | 66% | Calcifications are often seen as round bright images within the parenchyma or ducts. They are rare in PGs and more common in SMGs. Hyperechoic linear structures sometimes indicate calcification of a ligament. | 100% |
| **Posterior border visibility**  The posterior border of the PG and SMG is the deep gland boundary on transverse and longitudinal views. The posterior border is normally visible by ultrasonography. | 83% | The gland contour is normally identified by a hyperechoic line between the SGs and the adjacent tissues in normal conditions. | 100% |

SG, salivary gland; pSS, primary Sjögren’s syndrome; PG, parotid gland; SMG, submandibular gland

**#** Hypo/anechoic areas are among the abnormal echostructures of Homogeneity

*****Calcification is an abnormal echostructure of Echogenicity
