# Supplementary material for: Salivary gland ultrasound abnormalities in primary Sjögren’s syndrome: consensual US-SG core items definition and reliability
Source: RMD Open. 2017 Jun 9;3(1):e000364. doi: 10.1136/rmdopen-2016-000364 (PMC5575597; doi:10.1136/rmdopen-2016-000364)
Supplement: Supplementary data [file rmdopen-2016-000364supp002.pdf]

Ultrasonography of Parotid and submandibular glands in primary Sjögren Syndrome

A novel reference atlas for B-mode evaluation of parotid and submandibular glands

Sandrine Jousse-Joulin et. al

Included Salivary glands

- Parotid glands
- Submandibular glands

7 items:

- Echogenicity (normal or abnormal)
- Homogeneity and its related items:

1. Hyperechoic bands:
   - Presence of hyperechoic bands <20%
   - hyperechoic bands between 20%-50%
   - hyperechoic bands >50%
2. Hypo/anechoic area:
   - Isolated<25%
   - Localized 25-50%
   - Scattered
   - Diffuse>50%

- Lymph nodes
- Calcifications (present or not)
- Posterior border (visible or not)

PAROTID GLAND PROCEDURE

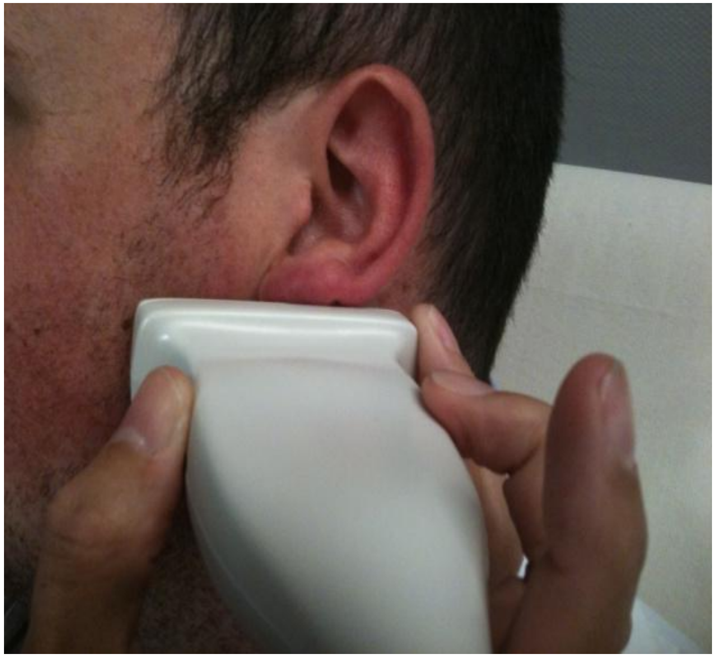

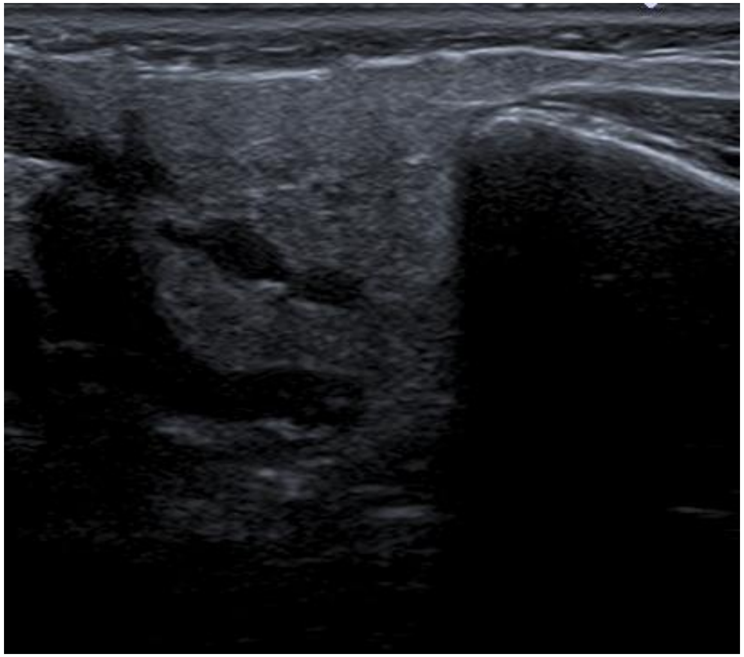

Parotid gland in axial scan

Jousse-Joulin al.
Ultrasonography scoring
reference atlas ver 1.0

PAROTID GLAND PROCEDURE

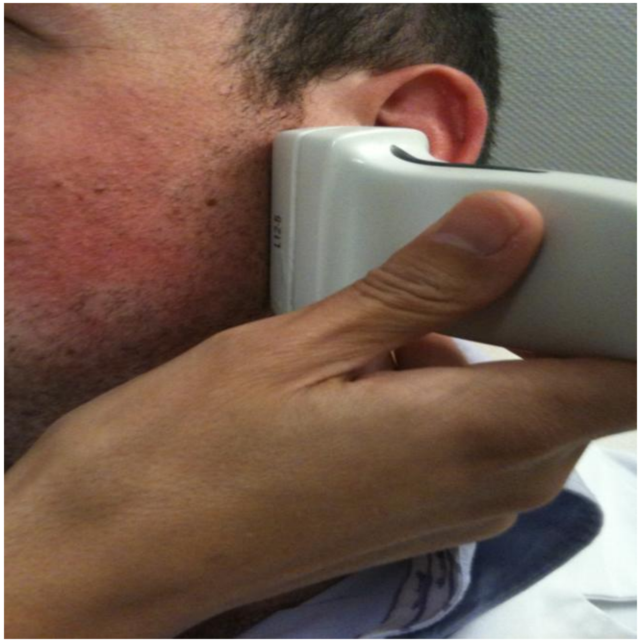

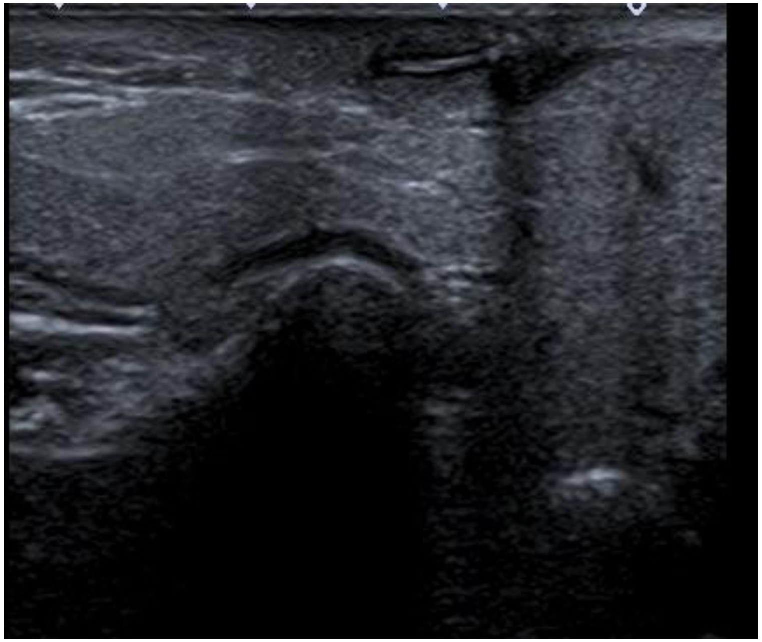

Parotid gland in longitudinal scan

Jousse-Joulin al.
Ultrasonography scoring
reference atlas ver 1.0

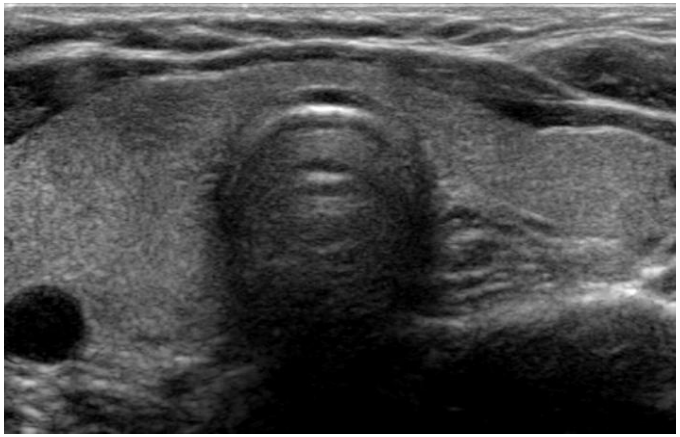

Thyroid parenchyma

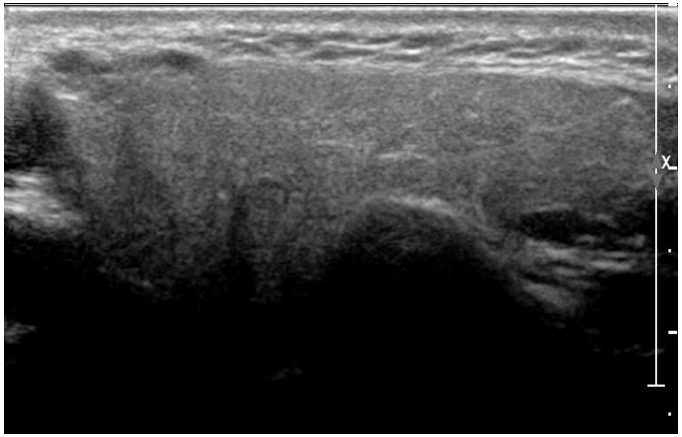

Parotid parenchyma

Normal echogenicity

Jousse-Joulin al.
Ultrasonography scoring
reference atlas ver 1.0

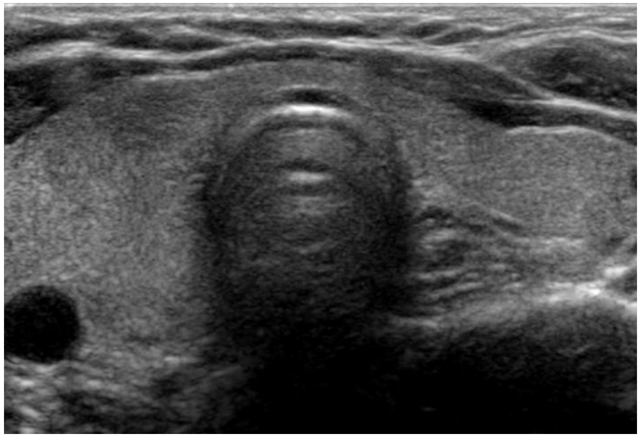

Thyroid parenchyma

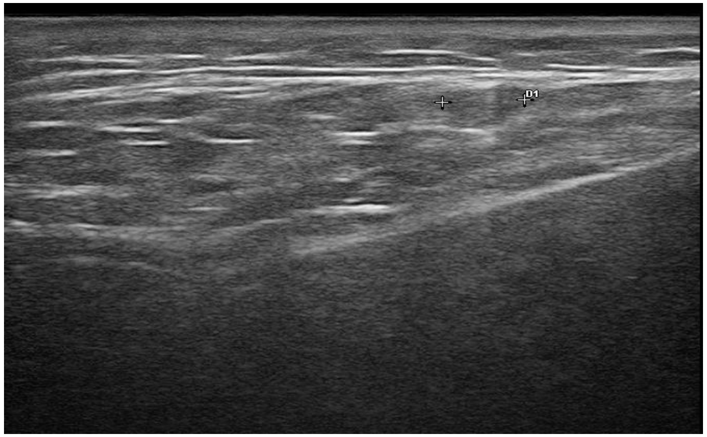

Parotid parenchyma

Abnormal echogenicity

Jousse-Joulin al.
Ultrasonography scoring
reference atlas ver 1.0

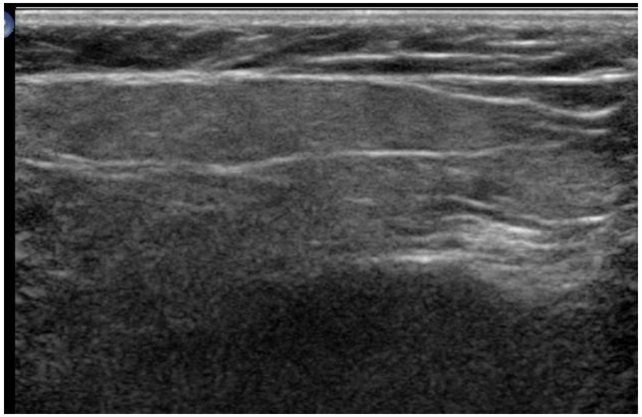

1

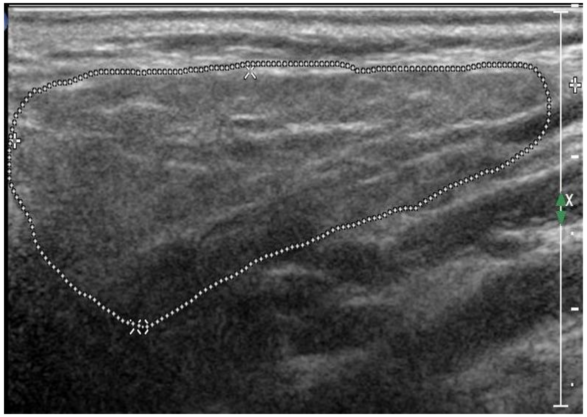

2

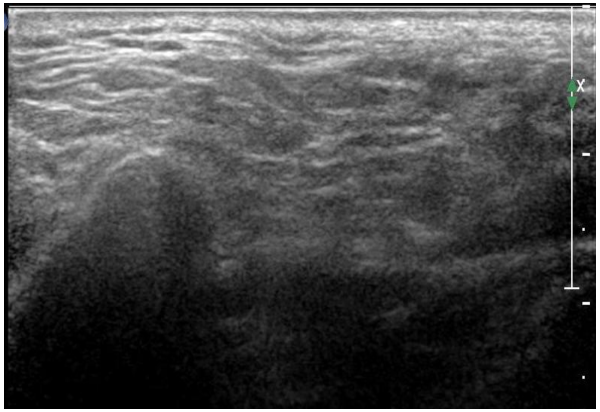

3

Hyperechoic bands in parotid gland parenchyma:

1. Less than 20% of the surface
2. Between 20-50% of the surface
3. More than 50% of the surface

Jousse-Joulin al.
Ultrasonography scoring
reference atlas ver 1.0

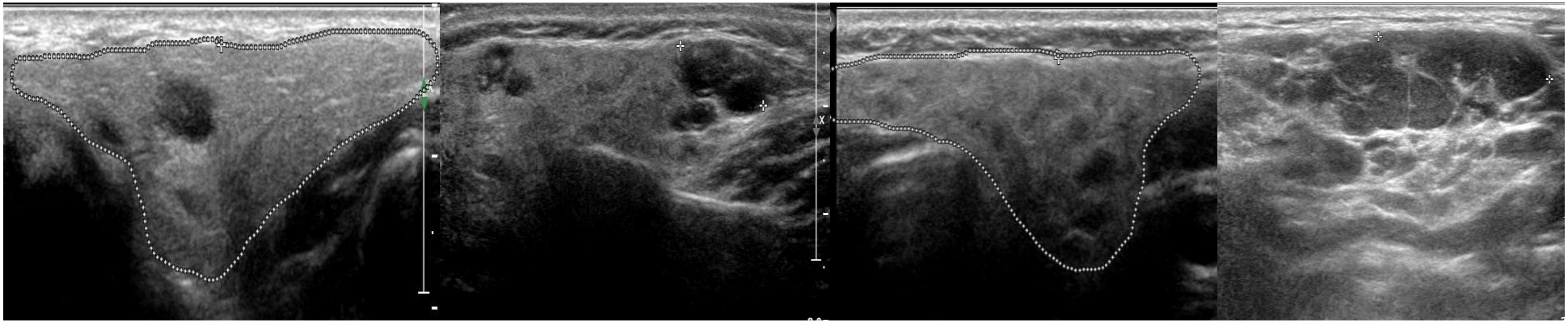

isolated localized scattered diffuse

Hypo/Anechoic areas diffuse in parotid parenchyma

Jousse-Joulin al.
Ultrasonography scoring
reference atlas ver 1.0

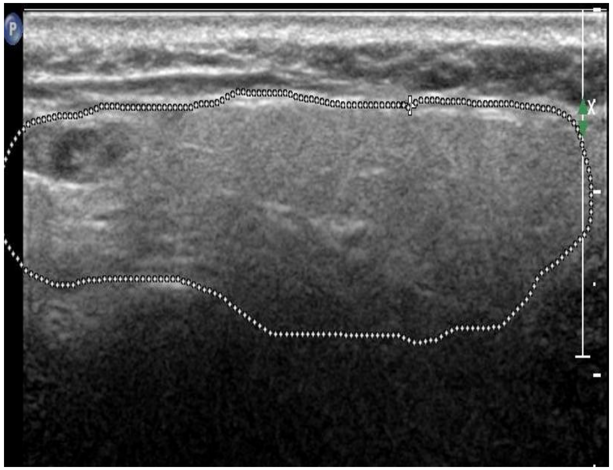

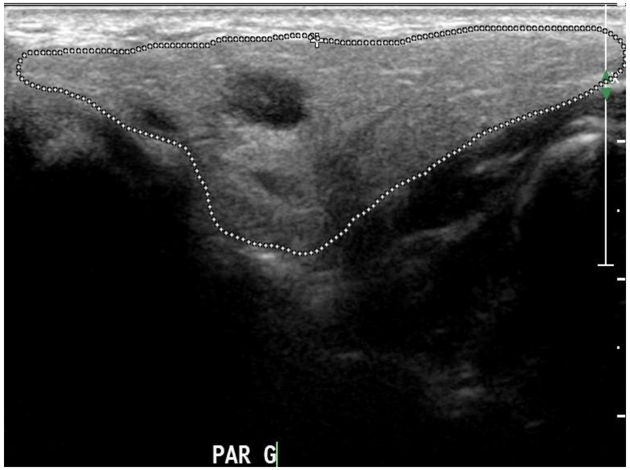

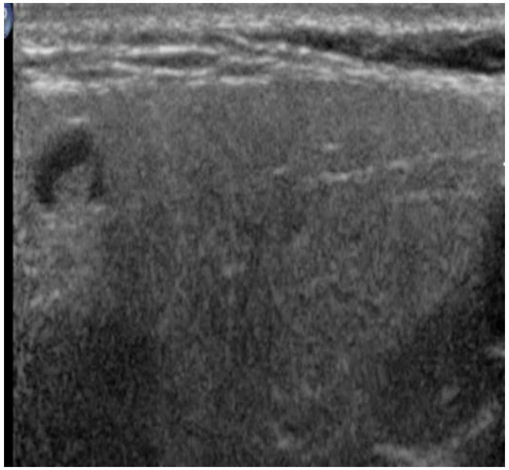

Normal lymph nodes in parotid gland

Jousse-Joulin al.
Ultrasonography scoring
reference atlas ver 1.0

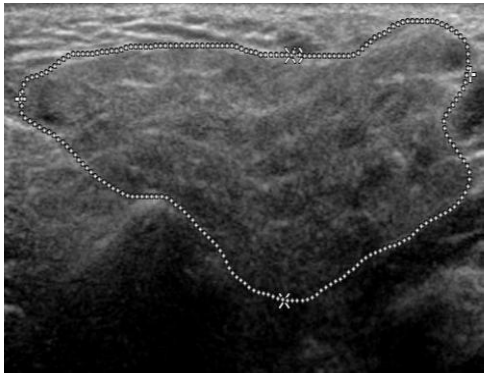

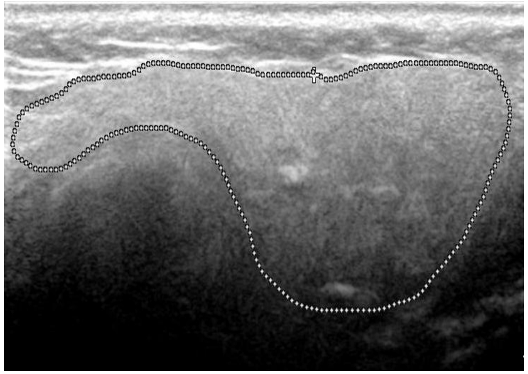

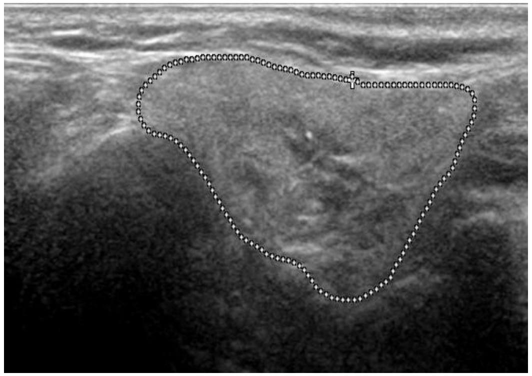

Calcifications in parotid gland

Jousse-Joulin al.
Ultrasonography scoring
reference atlas ver 1.0

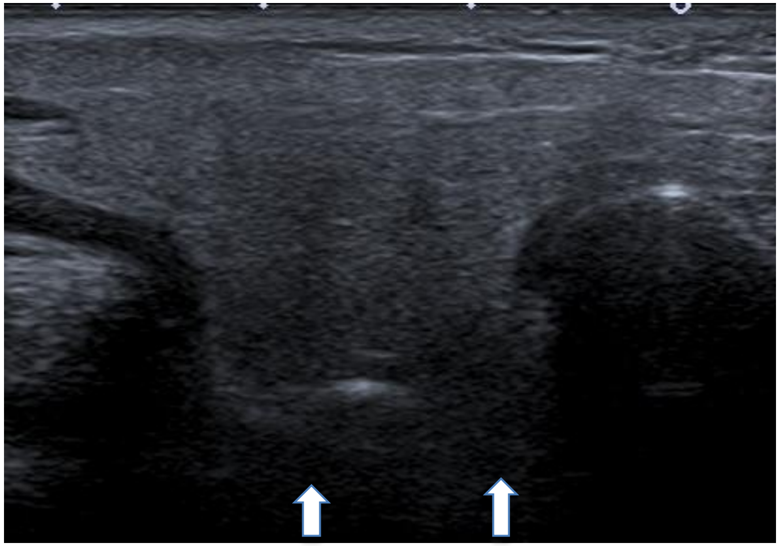

Axial scan

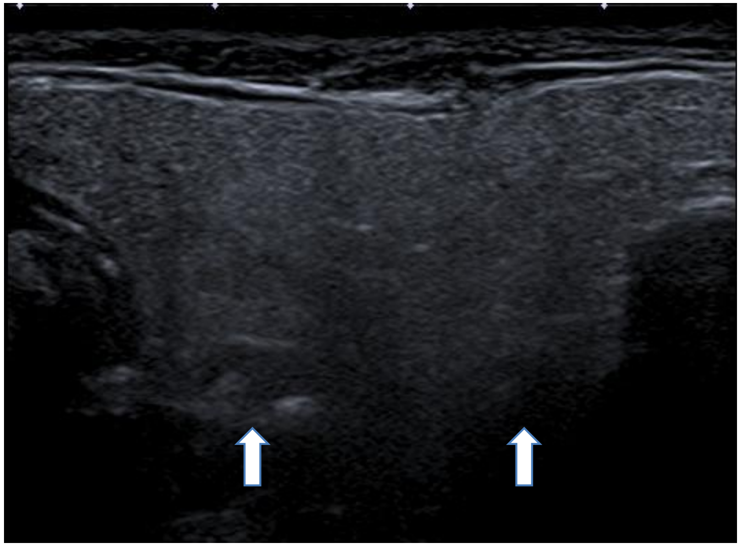

Longitudinal scan

Visible Posterior border in parotid gland

Jousse-Joulin al.
Ultrasonography scoring
reference atlas ver 1.0

SUBMANDIBULAR GLAND PROCEDURE

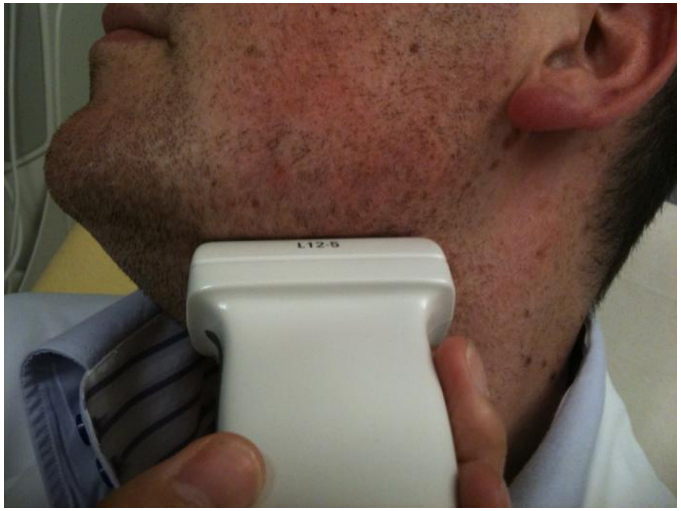

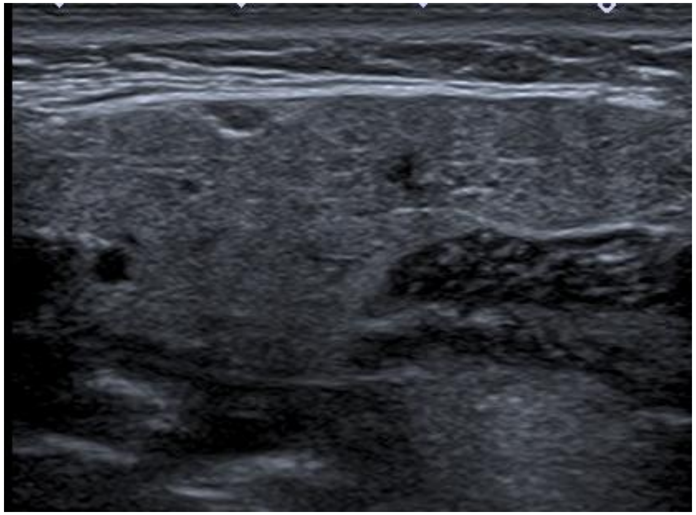

Jousse-Joulin al.
Ultrasonography scoring
reference atlas ver 1.0

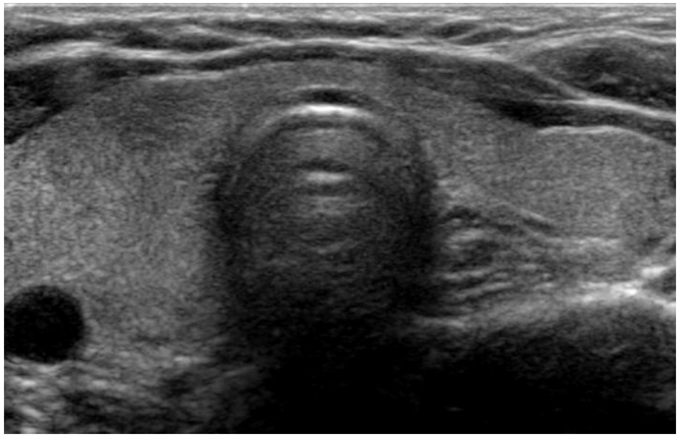

Thyroid parenchyma

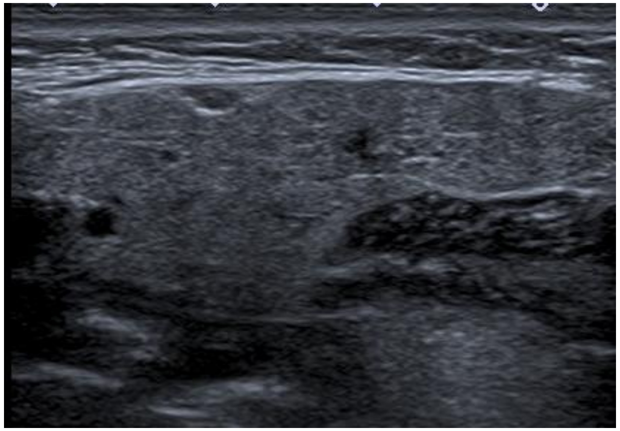

Submandibular parenchyma

Normal echogenicity

Jousse-Joulin al.
Ultrasonography scoring
reference atlas ver 1.0

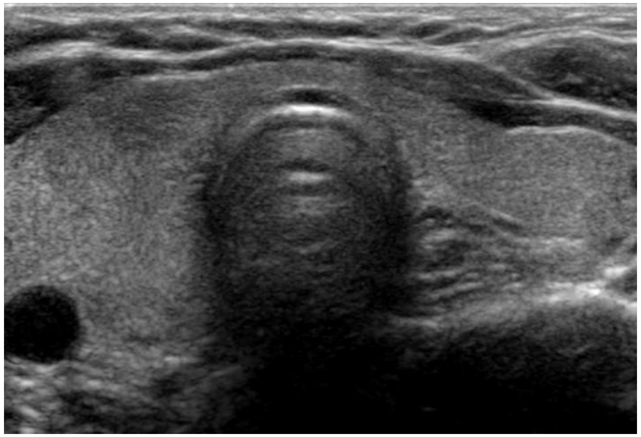

Thyroid parenchyma

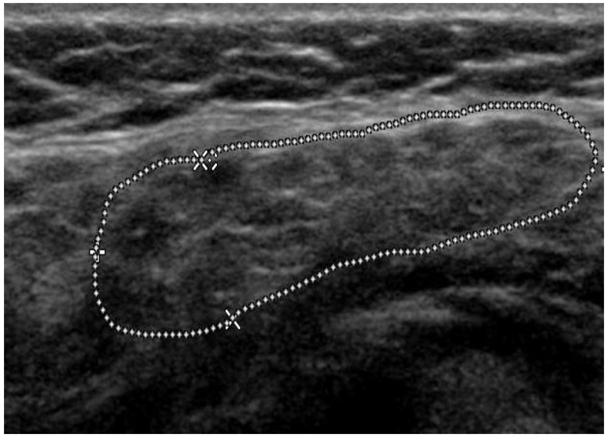

Submandibular parenchyma

Abnormal echogenicity

Jousse-Joulin al.
Ultrasonography scoring
reference atlas ver 1.0

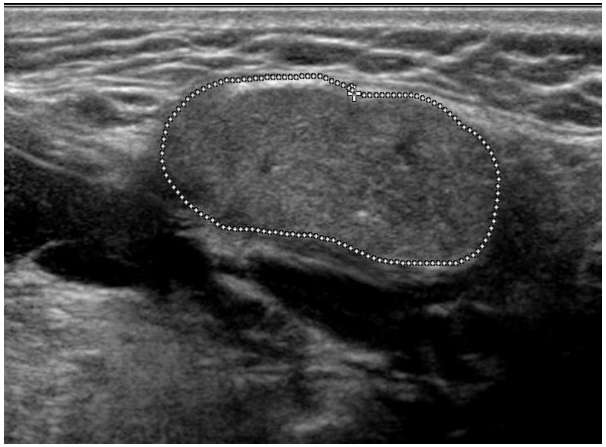

1

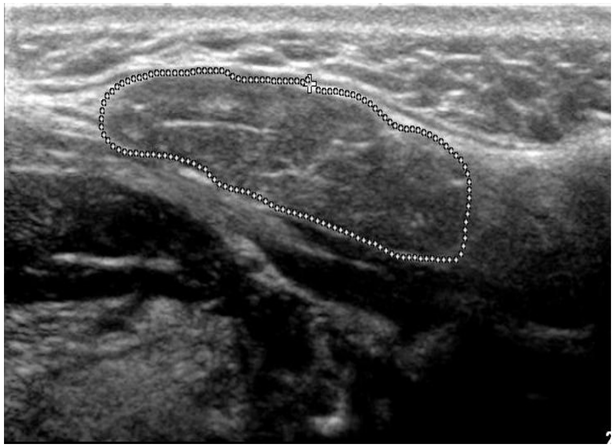

2

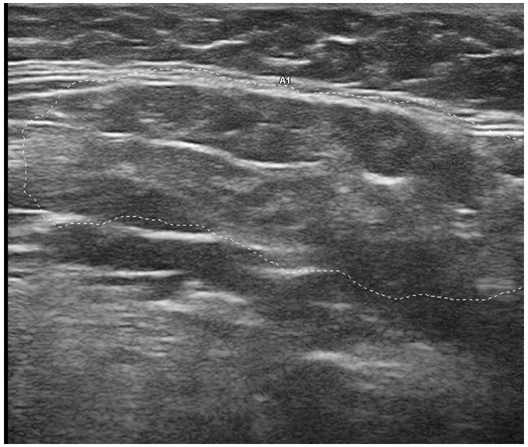

3

Hyperechoic bands in submandibular gland parenchyma:

1.Less than 20% of the surface

2.Between 25-50% of the surface

3.More than 50% of the surface

Jousse-Joulin al.
Ultrasonography scoring
reference atlas ver 1.0

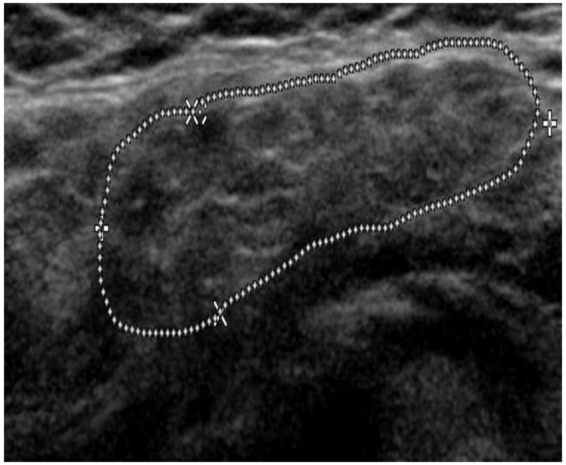

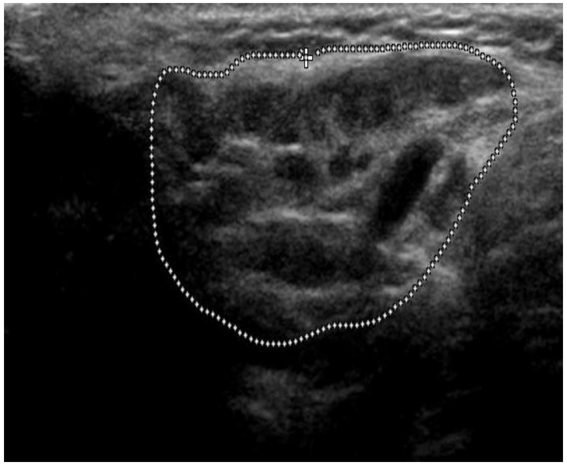

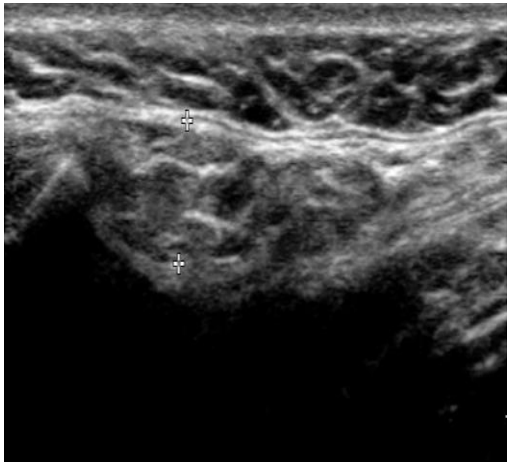

Hypo/Anechoic areas diffuse in submandibular parenchyma

Jousse-Joulin al.
Ultrasonography scoring
reference atlas ver 1.0

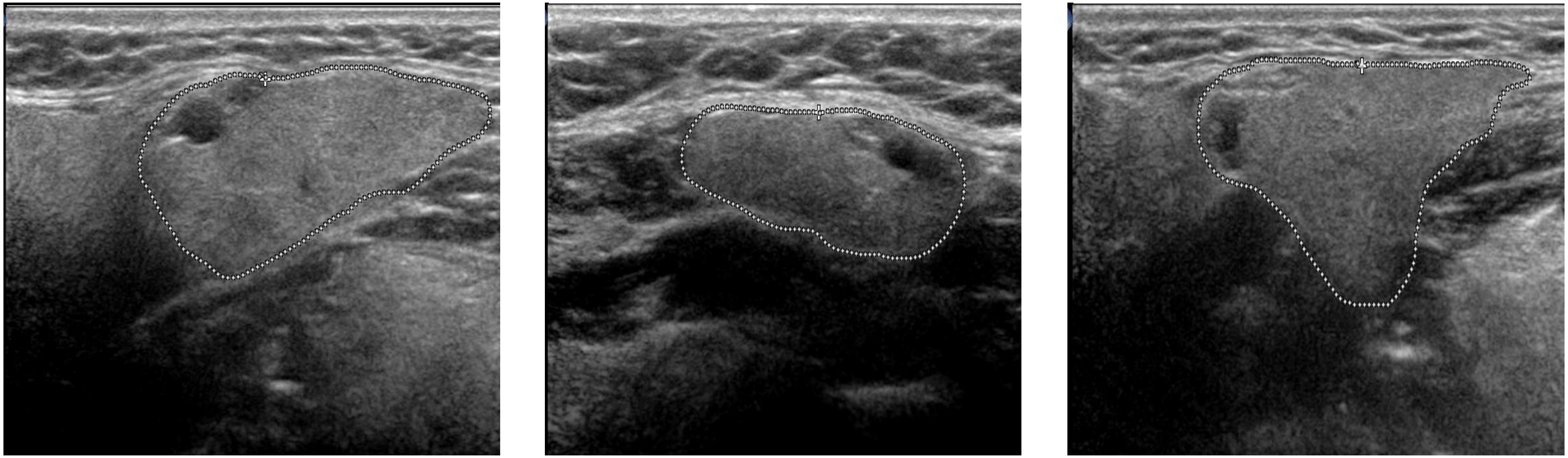

Hypo/Anechoic areas isolated in submandibular parenchyma

Jousse-Joulin al.
Ultrasonography scoring
reference atlas ver 1.0

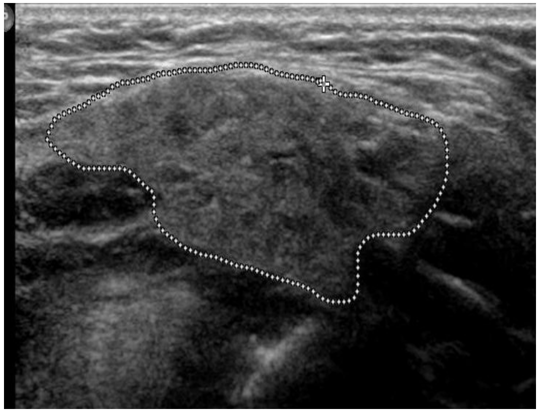 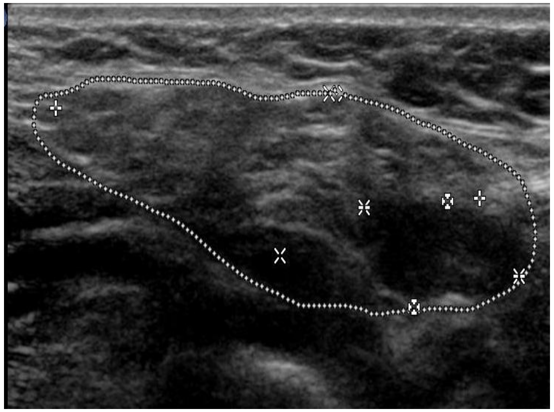 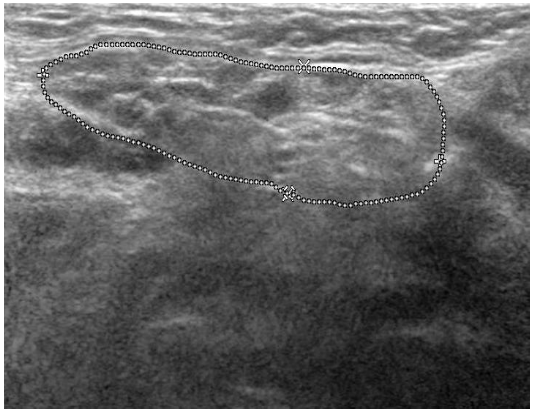

Hypo/Anechoic areas localized in submandibular parenchyma

Jousse-Joulin al.
Ultrasonography scoring
reference atlas ver 1.0

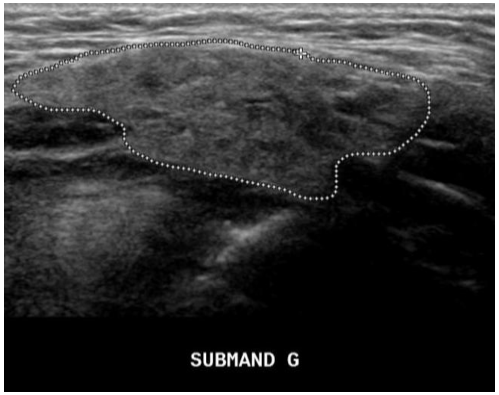

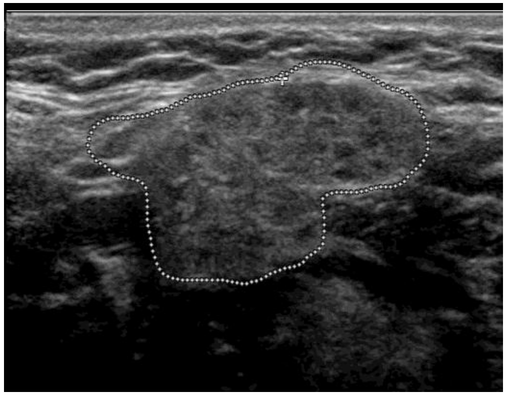
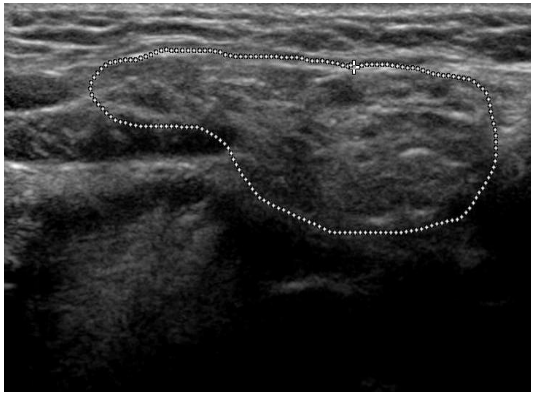

Hypo/Anechoic areas scattered in submandibular gland

Jousse-Joulin al.
Ultrasonography scoring
reference atlas ver 1.0

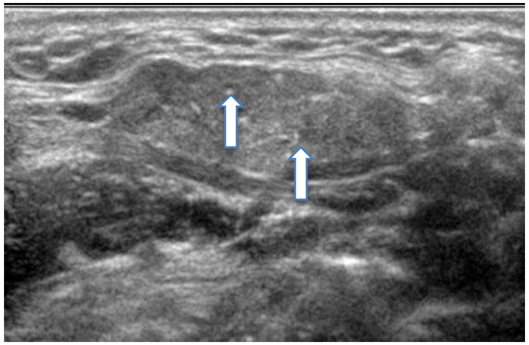

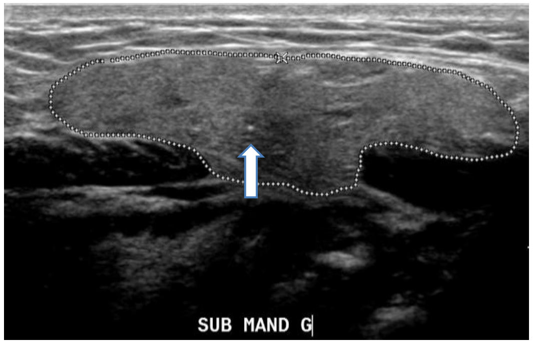

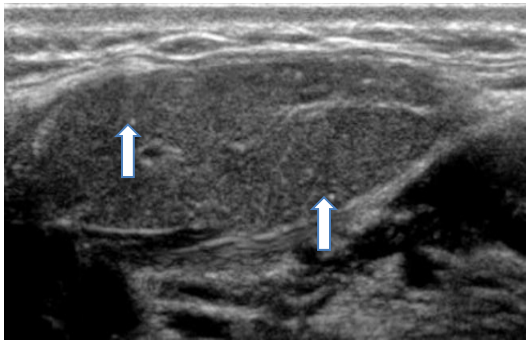

Calcifications in submandibular gland

Jousse-Joulin al.
Ultrasonography scoring
reference atlas ver 1.0

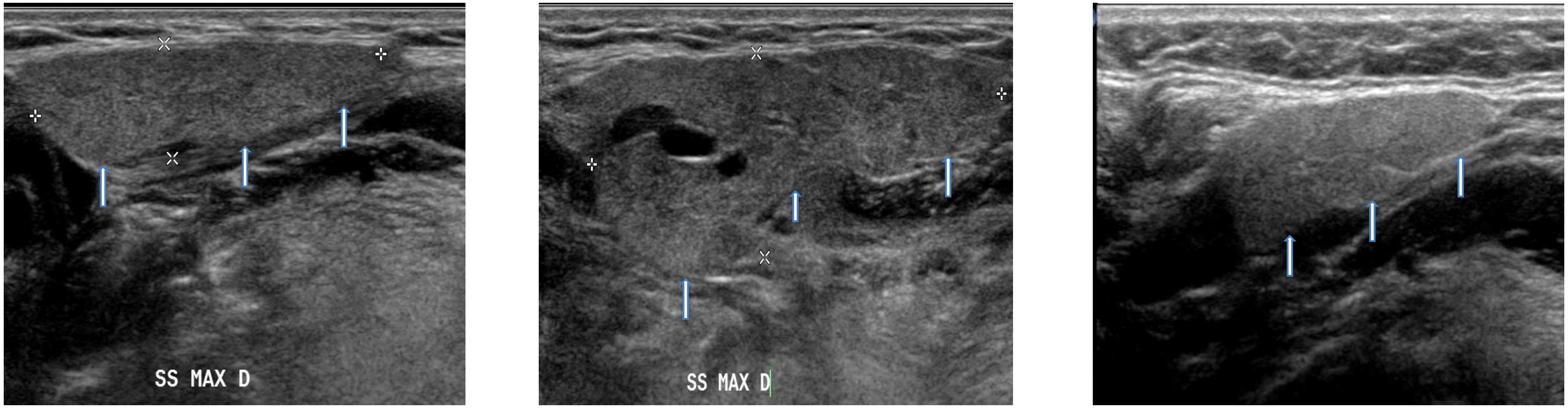

Visible posterior border in submandibular gland

Jousse-Joulin al.
Ultrasonography scoring
reference atlas ver 1.0
